# Deciphering CD4^+^ Naive T cell-mediated divergent pathogenic links between type 2 diabetes and pathologic scarring via an integrated multi-omics approach

**DOI:** 10.1097/JS9.0000000000004905

**Published:** 2026-01-19

**Authors:** Gehua Zhu, Jiamin Xu, Guanghua Guo, Feng Zhu

**Affiliations:** a Medical Centre of Burn Plastic and Wound Repair, The First Affiliated Hospital, Jiangxi Medical College, Nanchang University, China; b Department of Critical Care Medicine, Shanghai East Hospital, Tongji University School of Medicine, Shanghai, China

**Keywords:** hypertrophic scar, keloid, Mendelian randomization, single-cell RNA sequencing, type 2 diabetes

## Abstract

**Objective:**

This study aimed to investigate the causal relationship between diabetes mellitus and pathological scarring, including hypertrophic scars (HS) and keloids, and to elucidate the underlying immune cellular mechanisms and key molecular regulators.

**Methods:**

We employed a phenome-wide Mendelian randomization approach (MR-PheWAS) to identify potential causal links between diabetes and pathological scars. Separate Mendelian randomization meta-analyses (MR-Meta) were performed for type 1 diabetes (T1D) and type 2 diabetes (T2D). Integrative analyses incorporating single-cell RNA sequencing (scRNA-seq), cell–cell communication inference, metabolic pathway profiling, and transcriptomic differential expression and enrichment analyses were conducted to delineate scar heterogeneity mediated by diabetes-related cellular subpopulations and molecular drivers.

**Results:**

MR-PheWAS revealed significant causal associations between HS and multiple diseases. T2D exhibited a positive causal association with HS (OR: 1.13, 95% CI: 1.09–1.17, *P* < 0.0001), and a negative association with keloids (OR: 0.95, 95% CI: 0.92–0.99, *P* = 0.016). T1D showed no significant causal relationship with either scar type. Single-cell analysis identified an upregulation of CD4^+^ Naive T cells (CD4^+^ NT) in HS and a downregulation in keloids. This subset exhibited strong interactions with endothelial cells in both scar types, primarily enriched in the insulin signaling pathway (INS–INSR) signaling axis. Three pivotal genes (GPR35, TMEM91, and ZBTB32) were identified as overlapping molecular links between T2D and pathological scars, all expressed in CD4^+^ NT and showing inverse expression trends across HS and keloids. Pseudotime trajectory analysis further revealed divergent expression kinetics of these genes, characterized by early loss and late-phase upregulation. Metabolic pathway analysis implicated these genes in modulating metabolic reprogramming of the CD4^+^ NT population, thereby influencing scar differentiation.

**Conclusion:**

Convergent multi-omics evidence supports the hypothesis that T2D exerts opposing causal effects on different forms of pathological scarring, potentially mediated through CD4^+^ NT. The transcriptomic features, intercellular communication profiles, key gene signatures, and metabolic landscape of this subset collectively suggest that T2D may contribute to divergent fibrotic pathways in HS and keloids via immune microenvironmental modulation.

## Introduction

Wound healing is a highly coordinated and dynamic process that relies on a precise balance between inflammatory responses and tissue repair pathways. Disruption of this balance often results in the formation of pathological scars, primarily manifested as HS and keloids, both of which are characterized by excessive fibroblast proliferation and aberrant extracellular matrix (ECM) deposition^[1–3]^. Such scars commonly arise following dermal injuries – such as trauma, burns, surgical incisions, or insect bites[4] – and are driven by sustained local inflammation and dysregulated immune responses. HS typically remain confined within the original wound boundaries and may regress spontaneously over time, whereas keloids frequently invade surrounding normal skin and rarely resolve on their own. These lesions not only cause disfigurement but are also associated with pain, pruritus, contractures, and significant psychological distress[1]. Recent studies have identified multiple etiological contributors to pathological scarring, including chromosomal abnormalities[5], single nucleotide polymorphisms (SNPs)^[6,7]^, and epigenetic modifications^[8,9]^, as well as both systemic and local environmental risk factors^[10,11]^. Despite the availability of various therapeutic modalities – including surgical excision, laser therapy, radiotherapy, and intralesional injections – the long-term efficacy of current treatments remains unsatisfactory, and the clinical management of pathological scars continues to pose considerable challenges.

The interplay between diabetes mellitus and pathological scarring has recently emerged as a subject of considerable scientific inquiry. Effective skin repair is crucial for maintaining the body’s barrier function; however, dysregulated wound healing can result in either chronic non-healing ulcers or excessive fibrosis. Diabetes represents a chronic metabolic disorder defined by hyperglycemia and is often linked to low-grade inflammation, oxidative imbalance, and vascular microcirculatory impairment^[12,13]^, all of which may profoundly influence each stage of wound healing. Sustained elevations in blood glucose levels facilitate the non-enzymatic modification of proteins, lipids, and nucleic acids, ultimately resulting in the buildup of advanced glycation end-products (AGEs)[14]. These AGEs have been implicated in various diabetes-related complications, including chronic skin injury and impaired repair[15]. AGEs reduce collagen flexibility and solubility while also accelerating fibrogenesis and cutaneous aging processes[16].

In pathological scarring – HS and keloids – an expanding body of evidence implicates focal immune disequilibrium with pronounced T-cell infiltration and phenotypic remodeling, wherein immune–fibroblast crosstalk jointly drives collagen deposition and matrix remodeling; for example, the lesional dermis frequently shows enrichment of CD3^+^/CD4^+^ T cells coupled to fibroblast inflammatory–fibrotic signaling, and several studies suggest that Treg-associated signals may constrain profibrotic phenotypes^[17–19]^. In the context of diabetes, sustained hyperglycemia and metabolic imbalance reprogram adaptive immunity, deranging T-cell immunometabolic effector programs and contributing to multiple complications, including non-healing chronic wounds; recent reviews synthesize the key cellular and pathway aberrations and nominate putative immunologic targets in diabetic wounds^[20,21]^. Mechanistically, hyperglycemia and its stress derivatives activate canonical fibrogenic pathways (e.g., TGF-β/Smad), promoting aberrant fibroblast activation and excessive collagen deposition; concomitantly, diabetes-associated low-grade inflammation and immunosuppression disrupt the temporal orchestration of macrophage polarization and perturb macrophage–fibroblast interactions, thereby amplifying an inflammation–fibrosis positive feedback loop^[22–24]^. Guided by these priors, we center our investigation on the CD4^+^ NT subset – abundant within scar microenvironments and subject to dual metabolic and signaling control – as the conceptual entry point and rationale of this study; we also acknowledge the scarcity of large-scale prospective clinical evidence and definitive molecular causal chains, underscoring the need for integrated validation across large cohorts, single-cell multi-omics, and functional experimentation to delineate the regulatory nodes linking diabetes to pathological scarring and to inform therapeutic target development[25].

Residual biases often confound observational studies investigating the association between diabetes and HS or keloids, reverse causality, and survival effects, leading to inconsistent findings. To date, no definitive conclusions have been drawn regarding the causal relationship between diabetes and these scar phenotypes. Mendelian randomization (MR) infers causality by employing SNP-based instrumental variables (IVs), offering a rigorous genetic framework to disentangle exposure–outcome relationships. Significantly, MR minimizes confounding and limits the influence of reverse causality, thereby improving the robustness of causal conclusions. In this study, we integrated MR meta-analysis, single-cell analysis, and bulk transcriptomic analyses to comprehensively investigate the potential causal links between diabetes and pathological scarring. We incorporated type 2 diabetes (T2D) pancreatic islet scRNA-seq data to provide disease-conditioned immune–metabolic insights that may aid in elucidating the mechanisms underlying cutaneous scarring, rather than to assert cross-tissue equivalence. The islet data served solely as a conceptual anchor to assess whether alterations in INS–INSR signaling and T-cell metabolic programming align with the characteristics of HS and keloids. Given the inherent microenvironmental disparities and data heterogeneity, any observed concordance should be regarded as hypothesis-generating. Accordingly, we adopted a deliberately cautious tone throughout the manuscript. The report of this study was prepared in accordance with the TITAN guideline[26].

**HIGHLIGHTS**

- Type 2 diabetes exerts opposing causal effects on hypertrophic scars and keloids
- CD4^+^ Naive T cell metabolic reprogramming links metabolic dysregulation to scar heterogeneity
- Multi-omics integration identifies immune-metabolic targets for precision fibrosis intervention

## Materials and methods

### GWAS data sources for diabetes, hypertrophic scars, and keloids

The genome-wide association study (GWAS) data used in this study encompassed both type 1 diabetes (T1D) and T2D. T1D data were derived from five independent studies^[27–31]^ and the FinnGen consortium (https://www.finngen.fi/en), comprising a total of 37 244 cases and 1 281 458 controls. T2D data were aggregated from three studies within the DIAGRAM consortium^[32–34]^, nine large-scale cohort studies^[21,25,27,29–34]^, and the FinnGen database, totaling 366 980 cases and 1 830 428 controls (Supplemental Digital Content Table S1, available at: http://links.lww.com/JS9/G747). Genome-wide significant SNPs were selected using a threshold of *P* < 5 × 10^−8^. We implemented LD-based clumping to exclude correlated variants, setting the R^2^ to < 0.001 and the genomic span to 10 000 kb to retain independent instruments. Variants with ambiguous strand orientation or incompatible allelic pairs were excluded. Instrumental strength was quantified by the F-statistic (β^2^/SE^2^), and SNPs with F-statistics <10 were removed to avoid weak instrument bias. GWAS data for HS were sourced from the FinnGen consortium, involving a cohort of 467 741 individuals, including 2068 cases and 465 673 controls. Keloid-associated data were sourced from the study by Sakaue *et al*[31], involving 1732 cases and 658 915 controls.

### Mendelian randomization analysis

#### Univariable MR

To satisfy MR assumptions, all selected IVs were required to meet the following three criteria: (1) significant association with the exposure; (2) no direct association with the outcome; and (3) independence from confounding factors. Inverse variance weighted (IVW) analysis served as the primary MR method, while supplementary approaches (including MR-Egger regression, weighted median, and simple median) were applied to evaluate the robustness of the causal estimates. Heterogeneity among instruments was evaluated using Cochran’s Q test; significance was defined as *P* < 0.05. The MR-Egger intercept was used to detect horizontal pleiotropy, with a *P*-value > 0.05 indicating the absence of directional pleiotropic effects. Outlier SNPs were detected and adjusted using the MR-PRESSO approach, which accounts for horizontal pleiotropy through residual sum and outlier testing. After excluding outliers, sensitivity analyses were repeated to ensure the robustness of causal estimates.

**Figure 1.**
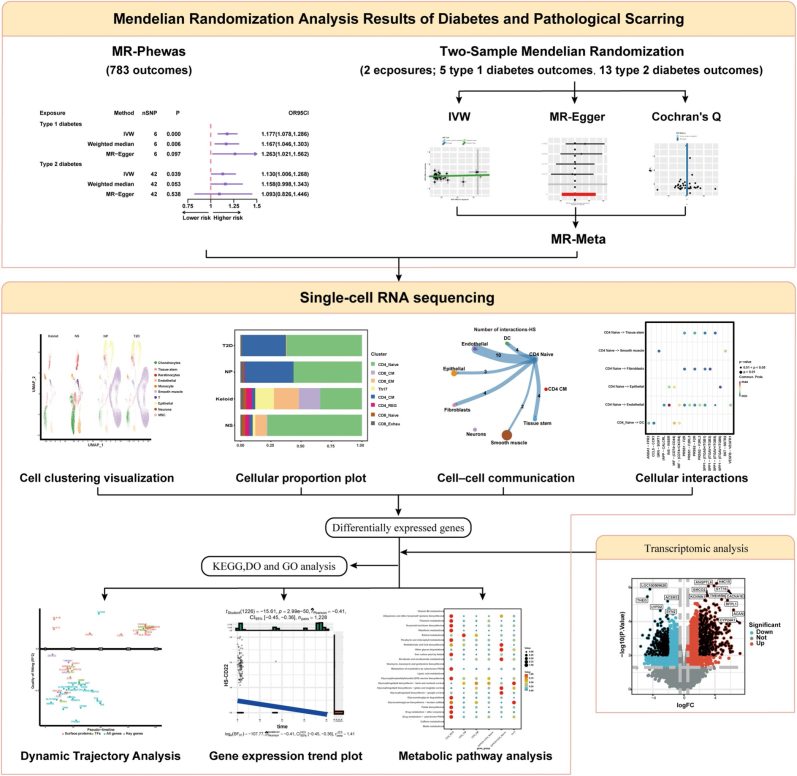
Flowchart of the study design.

To evaluate the presence of directional pleiotropy, funnel plots were constructed. To assess the influence of single variants, leave-one-out sensitivity testing was applied to examine the stability of causal inference (Fig. 1).

#### MR-PheWAS

MR phenome-wide association study (MR-PheWAS) was employed to systematically assess potential causal links between HS and a spectrum of other phenotypes. The analysis included 783 disease-related GWAS datasets from the UK Biobank SAIGE project (https://www.leelabsg.org/resources) as sources of exposure variables. Causal estimates were obtained using the IVW method and the Wald ratio. Tests for pleiotropy and heterogeneity, including Cochran’s Q test, MR-Egger intercept, and MR-PRESSO, followed the standard two-sample MR(TSMR) framework. All analyses were conducted in R version 4.1.3, primarily using the TwoSampleMR package (v0.5.6).

### Meta-analysis

Following the identification of 6 T1D-related and 13 T2D-related GWAS datasets for evaluating their respective causal associations with HS and keloids, we conducted a meta-analysis to integrate the results across individual studies. A random-effects model was employed to synthesize the TSMR estimates for HS and keloids, aiming to enhance statistical power and result robustness. We defined the primary outcome as the magnitude of association between diabetes and the development of HS or keloids, measured using odds ratios (ORs) and corresponding 95% CIs. The meta-analysis was based on IVW estimates derived from MR analyses, and potential publication bias was assessed using funnel plots. A two-sided *P*-value < 0.05 was considered statistically significant. All meta-analytical procedures were conducted in RStudio utilizing the “meta” package.

### Single-cell RNA sequencing analysis

The scRNA-seq datasets were retrieved from the Gene Expression Omnibus (GEO) repository (https://www.ncbi.nlm.nih.gov/gds). Data for normal skin (NS) and HS were obtained from GSE156326, while keloid data were derived from GSE181297 and GSE243716. Pancreatic islets from individuals with T2D and normal pancreatic islets (NP) were sourced from GSE221156. Detailed information regarding the number of samples, corresponding cell counts, and detected gene numbers for each dataset is provided in Supplemental Digital Content Table S2, available at: http://links.lww.com/JS9/G747. Before analysis, all datasets underwent batch effect correction and dimensionality reduction via principal component analysis (PCA).

#### Single-cell clustering and annotation

Single-cell analysis was performed using the Seurat R package[35] (v5.2.1). Raw PBMC sequencing data were converted into Seurat objects using the CreateSeuratObject function, followed by initial quality control based on sequencing metrics. Inclusion criteria were as follows: 200 ≤ nFeature_RNA ≤ 6500, nCount_RNA < 20 000, and mitochondrial gene content (percent.mt) < 15%. After filtering, the data were normalized and 2000 highly variable genes were identified for downstream analyses. Dimensionality reduction was conducted using principal component analysis (PCA). Nonlinear dimensionality reduction and visualization were performed using the RunUMAP function. Cell clustering was performed through the application of the FindNeighbors and FindClusters functions. Differential expression analysis was performed with a log fold change threshold of >0.5 using the FindMarkers function to identify cluster-specific marker genes.

Prior literature, the CellMarker database36, and automatic predictions from the SingleR package guided cell type annotation. A hybrid strategy combining manual curation and automated classification was applied to ensure accurate labeling of major cell types. Subsequently, secondary clustering was performed within the immune cell compartment to resolve finer subpopulations using the same analytical workflow.

#### Cell–cell communication analysis using cellchat

Following cell type annotation, intercellular communication between immune and non-immune cell types was systematically analyzed using the CellChat R package (v2.1.2) and the CellChatDB database (www.cellchat.org). This analysis quantitatively assessed ligand–receptor interactions across cell types, reconstructed the intercellular signaling network, and modeled the signal propagation dynamics within the tissue microenvironment.

#### Identification of key switch genes and dynamic expression analysis using GeneSwitches

To identify key regulatory genes involved in cell state transitions, the GeneSwitches R package (v0.1.0) was employed in conjunction with pseudotime analysis based on scRNA-seq data. Log-transformed gene expression matrices along defined pseudotemporal trajectories (from Monocle) were binarized using a threshold of 0.2. Logistic regression models were fitted to the binarized expression data to estimate gene-switching events and their corresponding transition time points and CIs. The quality of model fit was evaluated using pseudo R^2^ values, where R^2^ > 0 indicated upregulation and R^2^ < 0 denoted downregulation along the trajectory. High-confidence switching genes were visualized dynamically to capture their temporally ordered expression patterns during cellular transitions. Subsequently, metabolic pathway enrichment analysis was conducted for the identified switch genes.

#### Standardized single-cell validation workflow: decontamination, doublet detection, external re-annotation, and sensitivity analyses

Within the batch-corrected single-cell object, we first applied decontX to remove ambient RNA signals from the RNA counts and generated an RNA_decontX assay; positivity was then defined at two count-level thresholds (>0 and ≥2). Doublets were identified using scDblFinder, after which “gene-positivity × doublet” contingency tables were constructed and enrichment assessed by Fisher’s exact test. We next performed external re-annotation with SingleR using celldex reference panels and evaluated marker co-expression. Finally, sensitivity analyses were conducted.

### Transcriptomic analysis

To validate and further explore candidate genes and their mechanistic roles, bulk transcriptomic data (RNA-seq) were integrated into the analysis. Publicly available datasets GSE280420, GSE190135, and GSE188952 containing normal skin keloid and HS samples were retrieved from the GEO. To mitigate batch effects, the datasets were corrected using the Limma R package. Differential expression analysis of keloid samples in the GSE280420 dataset was conducted using thresholds of |log_2_FC| > 0.5 and *P* < 0.05. DEGs analysis in T2D using the GSE76895 dataset. Enrichment analyses were subsequently performed on the differentially expressed genes (DEGs), including Kyoto Encyclopedia of Genes and Genomes (KEGG) pathway and Gene Ontology (GO) functional annotation analyses.

## Results

### MR-PheWAS

**Figure 2.**
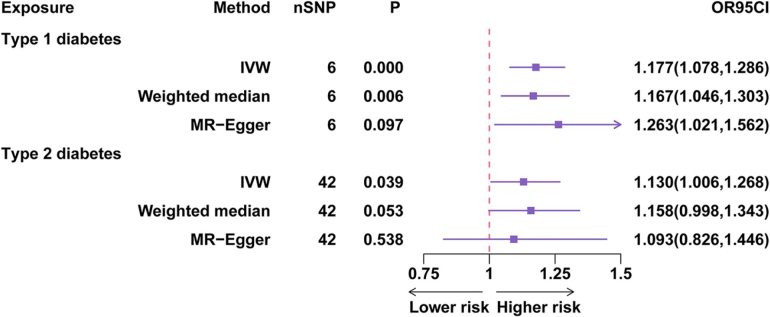
MR-PheWAS results of diabetes and hypertrophic scar. NSNP: number of single nucleotide polymorphism; IVW: inverse variance weighted; OR95CI: odds ratio with 95% confidence interval.

We first conducted an MR-PheWAS focused on HS. IVW estimates suggested potential causal associations between HS and several diseases, including T1D and T2D. However, none of these associations remained significant after multiple-testing correction (FDR > 0.05; Supplemental Digital Content Table S3, available at: http://links.lww.com/JS9/G747; Figure 2). We therefore treat these signals as hypothesis generating rather than confirmatory and do not assert a causal relationship between HS and T1D or T2D. No significant pleiotropy or heterogeneity was detected (Supplemental Digital Content Table S4, available at: http://links.lww.com/JS9/G747 and Supplemental Digital Content Table S5, available at: http://links.lww.com/JS9/G747), indicating that the selected IVs demonstrated good stability and validity.

### MR-meta analysis of diabetes and pathological scarring

#### Diabetes and hypertrophic scars

**Figure 3.**
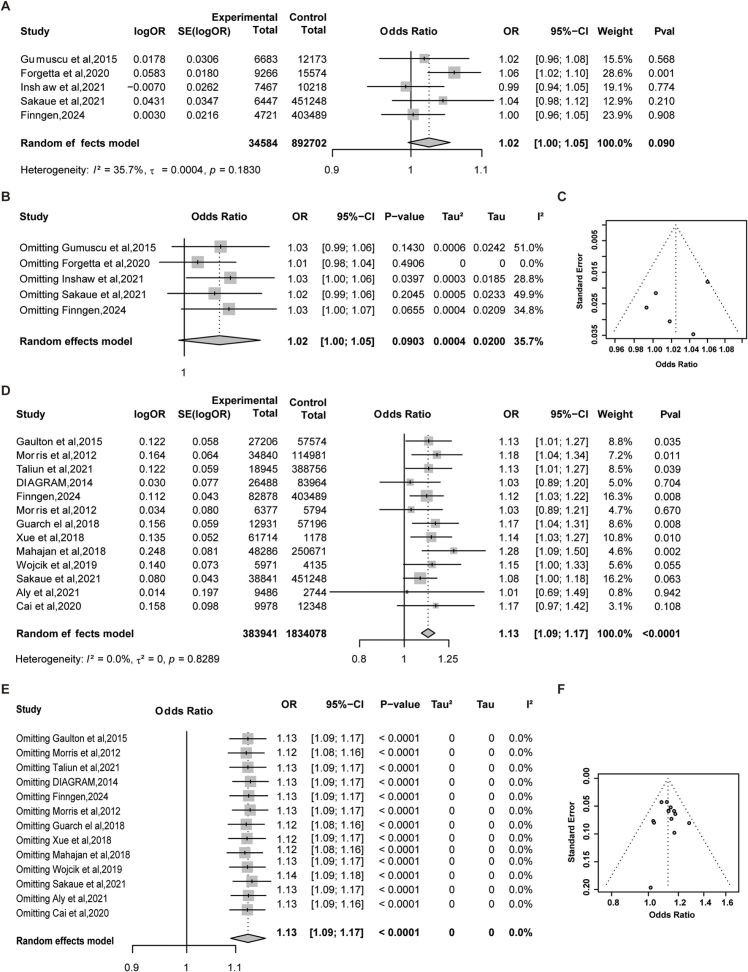
MR-Meta analysis results of diabetes and hypertrophic scar. (A) MR analysis results of T1D and HS; (B) Leave-one-out sensitivity analysis of T1D and HS; (C) Funnel plot of T1D and HS; (D) MR analysis results of T2D and HS; (E) Leave-one-out sensitivity analysis of T2D and HS; (F) Funnel plot of T2D and HS.

We conducted TSMR analyses between HS and five independent GWAS datasets related to T1D. All selected IVs were strong instruments (F-statistics > 10, Supplemental Digital Content Table S6, available at: http://links.lww.com/JS9/G747). However, the causal estimates across individual datasets were inconsistent, suggesting potential heterogeneity in the association between T1D and HS (Fig. 3A, Supplemental Digital Content Table S7, available at: http://links.lww.com/JS9/G747, Supplemental Digital Content Table S8, available at: http://links.lww.com/JS9/G747), and no significant pleiotropy was observed (Supplemental Digital Content Table S8, available at: http://links.lww.com/JS9/G747, Supplemental Digital Content Figure S1, available at:  http://links.lww.com/JS9/G797). Therefore, a meta-analysis of the TSMR results was performed. The MR-Meta analysis revealed no significant causal relationship between T1D and HS (OR: 1.02, 95% CI: 1.00–1.05, *P* = 0.090, Fig. 3A). Heterogeneity analysis indicated no significant inter-study variability (I^2^ = 35.7%, *P* = 0.1830, Fig. 3A). Leave-one-out sensitivity analysis and funnel plot inspection suggested no single study disproportionately influenced the overall effect size, supporting the robustness and lack of publication bias in the findings (Fig. 3B and C).

Subsequently, we performed TSMR analyses using 13 T2D-associated GWAS datasets as exposures and HS as the outcome. All IVs demonstrated strong instrument strength (Supplemental Digital Content Table S6, available at: http://links.lww.com/JS9/G747). The individual TSMR results showed variability across datasets (Fig. 3D, Supplemental Digital Content Table S7, available at: http://links.lww.com/JS9/G747), with no evidence of pleiotropy (Supplemental Digital Content Table S8, available at: http://links.lww.com/JS9/G747, Supplemental Digital Content Figure S2, available at:  http://links.lww.com/JS9/G797). Meta-analysis of the aggregated TSMR estimates indicated a significant positive causal association between T2D and HS, suggesting that T2D may promote the development of HS (OR: 1.13, 95% CI: 1.09–1.17, *P* < 0.0001, Fig. 3D). Heterogeneity testing revealed no significant inconsistency among studies (I^2^ = 0.0%, *P* = 0.8289, Fig. 3D). Leave-one-out sensitivity analysis supported the stability of this association, and funnel plot analysis showed no apparent publication bias (Fig. 3E and F), further affirming the robustness and reliability of the results.

#### Diabetes and keloids

**Figure 4.**
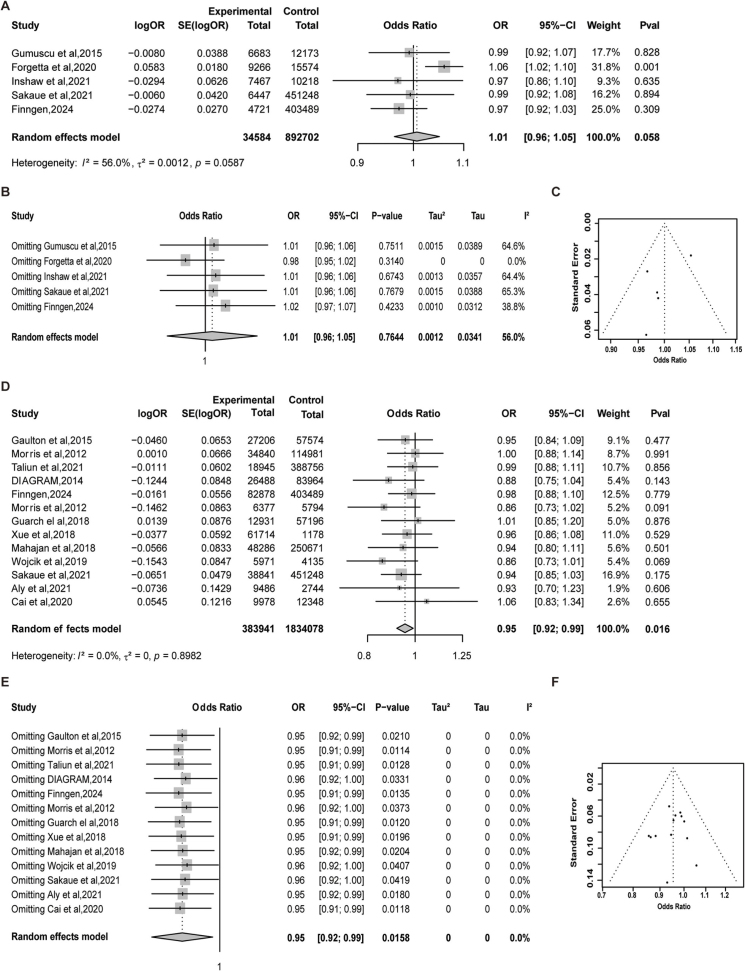
MR-Meta analysis results of diabetes and keloid. (A) MR analysis results of T1D and keloid; (B) Leave-one-out sensitivity analysis of T1D and keloid; (C) Funnel plot of T1D and keloid; (D) MR analysis results of T2D and keloid; (E) Leave-one-out sensitivity analysis of T2D and keloid; (F) Funnel plot of T2D and keloid.

To investigate the potential causal relationships between T1D, T2D, and keloids, TSMR analyses were conducted. All included IVs demonstrated strong instrument strength (F-statistics > 10, Supplemental Digital Content Table S9, available at: http://links.lww.com/JS9/G747). In the T1D–keloid analyses, causal estimates varied across datasets, indicating potential inter-study differences (Fig. 4A, Supplemental Digital Content Table S10, available at: http://links.lww.com/JS9/G747). The MR-Meta analysis showed no significant causal association between T1D and keloids (OR: 1.01, 95% CI: 0.96–1.05, *P* = 0.058, Fig. 4A). Leave-one-out sensitivity analysis indicated that no individual dataset disproportionately influenced the pooled effect estimate, supporting the stability and reliability of the meta-analytic result (Fig. 4B and C). In contrast, the MR-Meta analysis for T2D and keloids identified a statistically significant causal relationship, suggesting that T2D may play a suppressive role in keloid formation (OR: 0.95, 95% CI: 0.92–0.99, *P* = 0.016, Fig. 4D). Sensitivity testing and visual inspection of funnel plots confirmed the stability of the findings and revealed no substantial publication bias (Fig. 4E and F). No significant pleiotropy was detected (Supplemental Digital Content Table S11, available at: http://links.lww.com/JS9/G747, Supplemental Digital Content Figure S3, available at: http://links.lww.com/JS9/G797, Supplemental Digital Content Figure S4, available at: http://links.lww.com/JS9/G797).

### Single-cell clustering and annotation

MR-Meta analysis revealed no significant causal relationship between T1D and either HS or keloids. MR-Meta analysis revealed no significant causal associations between T1D and either HS or keloids. In contrast, T2D exhibited opposing and statistically significant causal effects on these two forms of pathological scarring: T2D was positively associated with an increased risk of HS, whereas it appeared to exert a potential protective effect against keloid formation. These findings suggest that T2D may differentially regulate scar pathogenesis through mechanistic heterogeneity.

**Figure 5.**
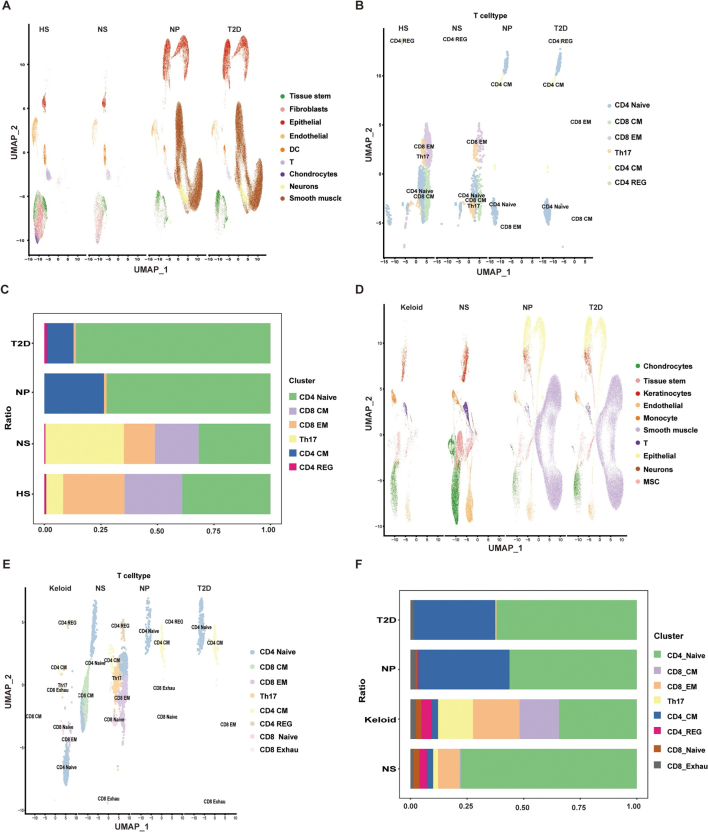
Filtering, processing, and T cell subset analysis of single-cell RNA sequencing data. (A) UMAP visualization of cell clustering based on marker genes in HS; (B) UMAP plot showing six T cell subpopulations identified by marker genes in HS; (C) proportional comparison of T cell subsets among T2D islets, normal islets, HS, and normal skin in HS; (D) UMAP visualization in keloid; (E) UMAP plot of T cell subpopulations in keloid; (F) proportional comparison of T cell subsets in keloid.

To elucidate the mechanistic basis of these differences, we integrated single-cell RNA-sequencing datasets spanning T2D, HS, and keloid cohorts, applied Harmony-based batch correction, and conducted downstream cell clustering and annotation. Following batch correction, inter-sample batch effects were markedly attenuated (Supplemental Digital Content Figure S5, available at: http://links.lww.com/JS9/G797). Multiple major cell types were identified, including fibroblasts, chondrocytes, tissue stem cells, neurons, epithelial cells, endothelial cells, dendritic cells, natural killer (NK) cells, T cells, smooth muscle cells, keratinocytes, and monocytes (Fig. 5A and D). Cellular profiling indicated substantial group-specific differences, with HS characterized by elevated T cell proportions and keloids showing a significant reduction relative to normal tissues. To explore this trend further, we performed subclustering and functional annotation within the T cell population (Fig. 5B and E). The results demonstrated a significant increase in CD4^+^ NT in HS compared to controls, while the same subset was markedly reduced in keloid samples.

### Cell–cell communication

**Figure 6.**
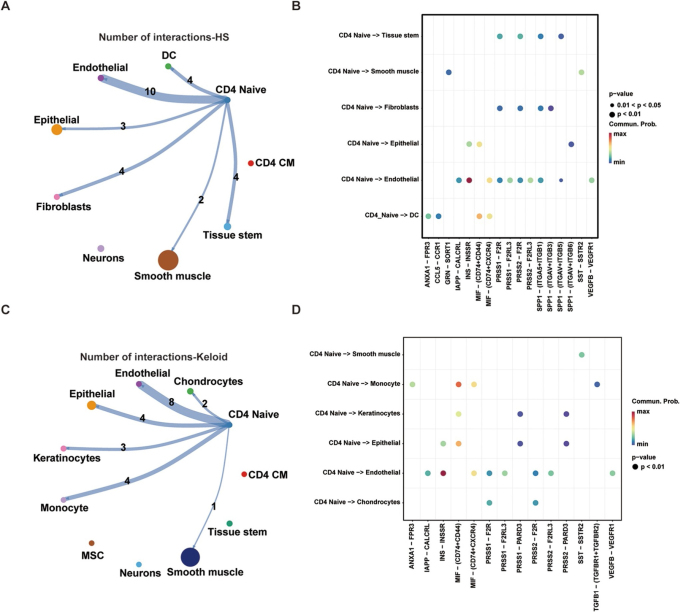
Communication and metabolic pathway analysis of CD4^+^ Naive T cells. (A) Interaction count analysis of the intercellular communication network in HS; (B) Bubble plot of ligand–receptor-mediated interactions between T cells and other cell types in HS; (C) Interaction count analysis of the intercellular communication network in keloid; (D) Bubble plot of ligand–receptor-mediated interactions between T cells and other cell types in keloid.

To further elucidate the microenvironmental regulatory roles of CD4^+^ NT in HS and keloids, we conducted a systematic analysis of their intercellular communication networks and ligand–receptor signaling pathways. In HS, CD4^+^ NT primarily interacted with smooth muscle cells, endothelial cells, and fibroblasts (Fig. 6A), suggesting their involvement in processes such as local angiogenesis, tissue remodeling, and mechanical tension regulation. In contrast, CD4^+^ NT in keloid tissues exhibited broader intercellular interactions, particularly with keratinocytes, monocytes, and chondrocytes (Fig. 6C), implicating their coordinated roles in inflammatory responses, aberrant epidermal proliferation, and extracellular matrix remodeling.

Pathway analysis revealed that in HS, CD4^+^ NT predominantly communicated with endothelial cells via the INS–INSR signaling axis, which exhibited the highest interaction probability. In keloids, these cells also showed strong interactions with monocytes and epithelial cells, and in addition to the INS–INSR pathway, the MIF–(CD74 + CD44) axis was also prominently enriched. Notably, the INS–INSR signaling consistently demonstrated the most significant and probable communication between CD4^+^ NT and endothelial cells in both scar types, indicating a shared role in vascular modulation. The enrichment of MIF–(CD74 + CD44) signaling in keloids suggests a heightened inflammatory state and implies that CD4^+^ NT participate in more complex immune–structural interactions within the keloid microenvironment.

### Identification of key genes

**Figure 7.**
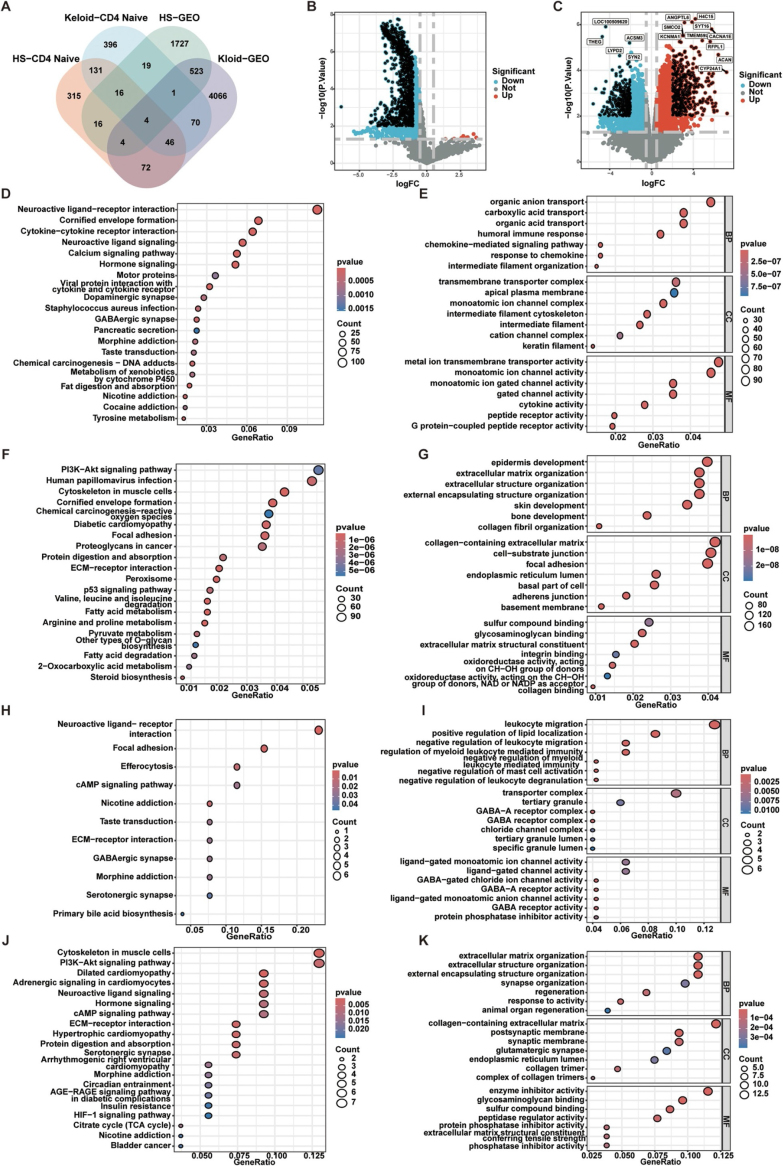
Identification of key genes and their pathway enrichment analysis. (A) Venn diagram identifying commonly differentially expressed key genes in both types of pathological scars; (B) Volcano plot of differentially expressed genes in hypertrophic scar; (C) Volcano plot of differentially expressed genes in keloid; (D and E) KEGG and GO analysis results of differentially expressed genes in the hypertrophic scar group; (F and G) KEGG and GO analysis results of differentially expressed genes in the keloid group; (H and I) KEGG and GO analysis result of DEGs at the T2D–hypertrophic scar intersection; (J and K) KEGG and GO analysis result of DEGs at the T2D–keloid intersection.

To investigate the molecular basis underlying the divergent effects of T2D on HS and keloids, we focused on T2D-associated CD4^+^ NT and analyzed their differential expression profiles in both scar types. Differential gene expression analysis identified 604 DEGs in HS and 683 DEGs in keloids. To isolate potential regulatory genes involved in both fibrotic processes but exhibiting directionally distinct roles, we performed an intersection analysis of the two DEG sets. A total of 197 shared DEGs were identified, suggesting that these genes may represent critical molecular nodes mediating the opposing effects of T2D in HS and keloid pathogenesis (Fig. 7A).

### Transcriptomic identification of differential genes and enrichment analysis

To further validate and dissect the molecular features associated with each scar phenotype, we systematically compared transcriptomic profiles from HS and keloid tissue samples. This analysis identified 2310 DEGs in HS (Fig. 7B, Supplemental Digital Content Table S12, available at: http://links.lww.com/JS9/G747) and 4786 DEGs in keloids (Fig. 7C, Supplemental Digital Content Table S13, available at: http://links.lww.com/JS9/G747). GO and KEGG enrichment analyses were subsequently performed to characterize their functional differences at the transcriptional level.

In HS, DEGs were enriched in pathways related to neurotransmission (e.g., neuroactive ligand–receptor interaction, dopaminergic synapse), cytokine signaling (cytokine–cytokine receptor interaction), and cytochrome P450-mediated drug metabolism (Fig. 7D), indicating that HS is predominantly associated with stress responses, inflammatory regulation, and wound repair mechanisms. Corresponding GO terms were enriched for ion transport, chemokine response, membrane architecture, and cytokine receptor activity (Fig. 7E), highlighting immune regulation centered on barrier integrity and inflammation control. In contrast, KEGG analysis of keloids revealed significant enrichment in the PI3K-Akt signaling pathway, ECM–receptor interaction, focal adhesion, and chemical carcinogenesis–reactive oxygen species (ROS) pathways (Fig. 7F), suggesting active extracellular matrix remodeling, oxidative stress response, and enhanced cell motility. Metabolic pathway enrichment was also prominent, including fatty acid metabolism, amino acid degradation, and energy metabolism, reflecting a metabolically active state. GO analysis further revealed that keloid tissues were significantly activated in biological processes related to skin, skeletal, and epidermal development; enriched for cellular components such as collagen fibers, extracellular matrix attachments, and adhesion complexes; and associated with molecular functions such as glycosaminoglycan binding, enzymatic catalysis, and oxidoreductase activity (Fig. 7G). These findings collectively indicate that keloid formation is accompanied by sustained structural remodeling and aberrant energy–metabolic regulation, manifesting a highly fibrotic and metabolically active pathology.

The results further revealed significant enrichment of signaling pathways closely associated with ROS, including Chemical carcinogenesis – ROS and Oxidative phosphorylation. In parallel, the enrichment of the VEGF signaling pathway underscores its pivotal role in angiogenesis. Moreover, the enrichment of pathways such as ECM–receptor interaction, focal adhesion, and proteoglycans in cancer/glycosaminoglycan biosynthesis reflects aberrant extracellular matrix deposition, fibrotic remodeling, and fibroblast adhesion and migration, all of which are intricately linked to the mechanisms of scar formation. Notably, PI3K/Akt, VEGF, and cytokine receptor related pathways show concordant enrichment with multiple energy and substrate metabolism pathways, including glycolysis, fatty acid metabolism, oxidative phosphorylation, and redox regulation. This pattern suggests an immune receptor signaling driven immune metabolic reprogramming axis that may modulate the effector threshold of T cells, fibroblast activity, and matrix deposition, thereby coupling immune responses with metabolic supply and demand. This immune metabolic axis is corroborated by subsequent pathway and single cell analyses and provides a mechanistic basis for the later interpretation of CD4^+^ NT metabolic phenotypes and their divergent impacts on HS and keloids.

Building on these findings, to further delineate the molecular basis underlying the opposing effects of T2D on HS and keloids, we intersected DEGs derived from HS and keloid tissues with DEGs from T2D skin transcriptomic profiles, thereby generating “T2D–HS” and “T2D–keloid” intersecting gene sets, which were subsequently subjected to GO and KEGG enrichment analyses. The “T2D–HS” intersecting gene set was predominantly enriched in pathways related to focal adhesion, ECM–receptor interaction, efferocytosis, and neuroactive ligand–receptor/cAMP signaling; GO terms were mainly associated with leukocyte migration, regulation of myeloid immune responses, tertiary granule and mast-cell degranulation, as well as GABA(A) receptor and chloride channel activity, collectively indicating a more pronounced transcriptional signature linked to local tissue repair and immune cell trafficking (Fig. 7H and I). In contrast, the “T2D–keloid” intersecting gene set was significantly enriched in metabolic and hypoxia-associated pathways, including PI3K–Akt signaling, insulin resistance, AGE–RAGE signaling, and HIF-1 signaling. GO analysis further highlighted robust enrichment of extracellular matrix/structure organization, assembly of endoplasmic reticulum–localized collagen trimers, glycosaminoglycan binding, and regulation of protease activity, together with synapse organization–related terms (Fig. 7J and K), suggesting that the T2D–keloid axis is preferentially characterized by a metabolism–matrix remodeling–coupled transcriptional program.

To further identify genes potentially mediating the differential effects of T2D in HS and keloids, we intersected the transcriptomic DEG sets from both scar types with the 197 DEGs identified in CD4^+^ NT. This integrative analysis identified four candidate key regulatory genes: CD22, GPR35, TMEM91, and ZBTB32 (Fig. 7A). Among them, CD22, GPR35, and ZBTB32 were significantly downregulated in HS (logFC < 0) and upregulated in keloids (logFC > 0), exhibiting completely opposite expression trends between the two scar types.

### Re-evaluation of CD22 signal following decontamination and doublet correction

Given that CD22 is predominantly expressed on B cells rather than T cells, we re-evaluated this signal. Within the CD4^+^ NT cluster, decontX-denoised counts revealed a sporadic CD22 transcript frequency of 0.34% (3/888); under a more stringent threshold (counts ≥ 2), the frequency was 0.115% (1/888). Doublet detection with scDblFinder showed no enrichment of CD22 positivity among doublets (Fisher’s exact test *P* = 1.0; Supplemental Digital Content Table S14, available at: http://links.lww.com/JS9/G747). Consistent with these statistics, a 2 × 2 contingency heatmap demonstrates that CD22 >0 cells do not cluster on the doublet axis (Supplemental Digital Content Figure S6A, available at: http://links.lww.com/JS9/G797). Per-cell bar plots indicate that only the humanscar3 cell exhibits a B-lineage co-expression fingerprint (MS4A1, CD79A, CD74, BANK1; Supplemental Digital Content Figure S6B, available at: http://links.lww.com/JS9/G797), whereas the ND1 and TD3 cells lack this signature (Supplemental Digital Content Table S15, available at: http://links.lww.com/JS9/G747), supporting B-like misclassification/residuals for the former and sporadic ambient RNA reads for the latter. UMAP highlighting further shows that CD22 >0 cells appear as isolated points without local aggregation or a discrete subcluster (Supplemental Digital Content Figure S6C, available at: http://links.lww.com/JS9/G797). Taken together, CD22 does not constitute a stable expression feature of CD4^+^ NT in our dataset.

### Dynamic trajectory analysis of key genes during disease progression

**Figure 8.**
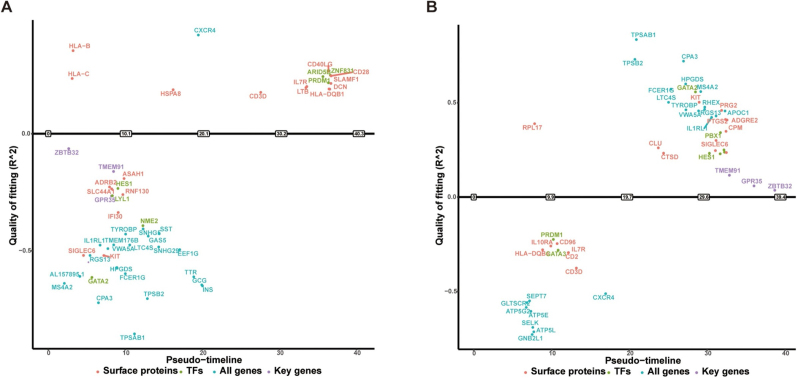
Expression trajectories of key genes along the pseudotime axis. (A) HS; (B) keloid.

To further elucidate the fate-determining processes of key genes in CD4^+^ NT, we employed the GeneSwitches algorithm to model the activation and silencing dynamics of three candidate genes (GPR35, TMEM91, and ZBTB32) along the pseudotime trajectory. This analysis aimed to predict the temporal expression patterns of these genes during cellular state transitions (Fig. 8). The results revealed striking differences in gene activation timing between HS and keloids. In HS-derived CD4^+^ NT, these key genes were predominantly activated during the early pseudotime stages and rapidly silenced thereafter, suggesting their involvement in early immune-regulatory events. Conversely, in keloid-derived cells, the same gene set was gradually activated during the mid-to-late stages of pseudotime, implicating potential roles in tissue remodeling, metabolic adaptation, or the persistence of chronic inflammation. Furthermore, we plotted gene expression trends across pseudotime, which demonstrated a continuous downregulation of all three genes in HS. In contrast, in keloids, two of the three genes (*GPR35* and *ZBTB32*) exhibited marked upregulation at later pseudotime stages, with *TMEM91* showing no significant change (Supplemental Digital Content Figure S7, available at: http://links.lww.com/JS9/G797, Supplemental Digital Content Figure S8, available at: http://links.lww.com/JS9/G797). This expression pattern was consistent with the trends observed in the transcriptomic analysis, reinforcing the notion that key genes undergo divergent fate decisions in distinct scar types.

### Metabolic profiling of key genes during disease progression

**Figure 9.**
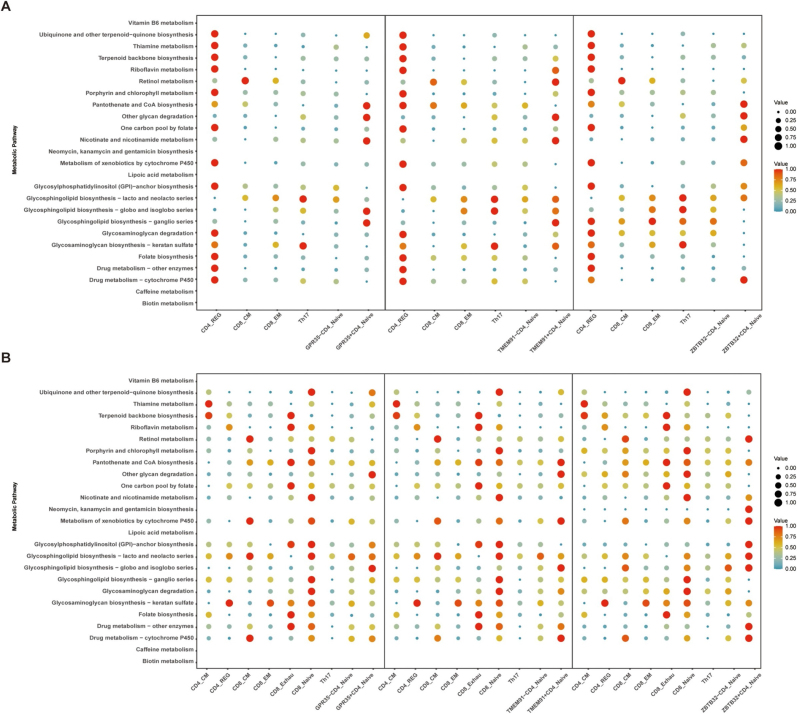
Single-cell metabolic pathway analysis. (A) HS; (B) keloid.

Single-cell metabolic profiling revealed environment-specific, pathway-cluster–level “shifts” in CD4^+^ NT between HS and keloids. CD4^+^ NT in HS exhibited prominent enrichment in coenzyme–mitochondrial modules, including ubiquinone/quinone, riboflavin, thiamine, pantothenate–CoA/lipoic acid, nicotinate–nicotinamide, and folate–one-carbon metabolism, indicating an oxidative phosphorylation (OXPHOS)/redox-prepared state with a lower metabolic activation threshold. Conversely, membrane glycolipid remodeling pathways were less enriched (Fig. 9). In contrast, in keloids, these cells were preferentially enriched in glycosphingolipid and GPI-anchor biosynthesis pathways, as well as glycosaminoglycan-related metabolism, accompanied by enhanced xenobiotic/drug metabolism (CYP) activity. This pattern suggests a metabolic orientation toward membrane structural remodeling and glycosylation, facilitating matrix–immune crosstalk, sustained inflammation, and fibrosis (Fig. 9). These cross-gene, cluster-level, and directionally consistent differences indicate that CD4^+^ NT may undergo metabolic reprogramming in the two scar types. This pattern aligns with transcriptomic pathway enrichment results, suggesting that metabolic remodeling underlies multiple layers of pathological scar formation and highlights potential targets for therapeutic interventions aimed at modulating T-cell metabolism.

### AUCell scoring and group comparison of pathway signatures in CD4^+^ Naive T cells

We added AUCell-AUC scoring for expanded IFNG/IL4/IL17A/IL10 signatures within CD4^+^ NT and performed sample-level Wilcoxon tests with BH-FDR correction. Keloids showed higher IL-4/Th2 activity than HS (*P* = 0.08), with a smaller concordant trend for IFN-γ/Th1; IL-17A and IL-10 showed no significant differences (Supplemental Digital Content Figure S9, available at: http://links.lww.com/JS9/G797, Supplemental Digital Content Table S16, available at: http://links.lww.com/JS9/G747). Given the limited sample size and sparsity intrinsic to scRNA-seq, none of these comparisons survived multiple testing. We accordingly frame them as suggestive trends/hypothesis-generating in the revised text and discuss the need for larger cohorts and functional validation in the Limitations section.

## Discussion

This study provides a comprehensive and integrative investigation combining MR with multi-omics approaches to systematically elucidate the putative causal relationships and molecular immune regulatory mechanisms linking T2D with HS and keloids. MR indicates a positive causal association between T2D and HS, whereas a negative association was observed for keloids, highlighting a strikingly divergent regulatory effect. The single-cell analysis further suggests that T2D modulates the composition and functional state of CD4^+^ NT, thereby reshaping the immune microenvironment within scar tissues. Cell–cell communication analyses additionally highlight INS–INSR signaling as a central axis mediating crosstalk between immune and stromal compartments in both scar types.

By integrating CD4^+^ NT-specific DEGs from single-cell datasets and bulk transcriptomic data, we identified three key genes – GPR35, TMEM91, and ZBTB32. These genes exhibited opposite expression patterns in HS and keloids, suggesting their involvement in distinct molecular subtypes and scar-specific pathogenic mechanisms. Metabolic pathway analysis further demonstrated that these genes are associated with a hyperactivated metabolic program in keloid-derived CD4^+^ NT, potentially contributing to pathological fibrosis through extracellular matrix remodeling and chronic inflammation maintenance. Collectively, this study delineates a multilayered framework – from genetic causality and single-cell immune landscapes to metabolic reprogramming – that underpins the differential regulatory effects of T2D on distinct types of pathological scarring. Our findings highlight metabolic dysregulation in CD4^+^ NT as a potential driver of aberrant scar formation and provide a theoretical foundation and therapeutic insight for targeted interventions in scar-related disorders.

Moreover, MR-PheWAS analysis revealed significant associations between HS and a range of metabolic and inflammatory conditions. Notably, MR-Meta analysis, for the first time from a genetic causality perspective, demonstrated that T2D exerts a promotive effect on HS formation while exhibiting a suppressive impact on keloid development. This finding addresses the limitations of previous epidemiological studies that relied solely on observational data, substantially strengthening the reliability and interpretability of causal inference. In contrast, T1D showed no significant causal association with either HS or keloids, underscoring fundamental differences between T1D and T2D in metabolic disturbance, insulin signaling, and immune regulation, which may underlie their divergent impacts on scar pathogenesis. This aligns with prior clinical observations that T2D is often accompanied by delayed wound healing, excessive fibrosis, and hypertrophic scarring^[36,37]^. However, previous studies were predominantly limited by small cohorts and lacked genetic and mechanistic validation. Through multi-omics integration, our study robustly confirms and expands upon the heterogeneous relationship between diabetes and pathological scarring, offering valuable mechanistic insights and a solid basis for future precision intervention strategies.

Single-cell analysis revealed a significant increase in the proportion of CD4^+^ NT in HS, whereas a marked reduction was observed in keloid tissues. This contrasting trend suggests that CD4^+^ NT may serve as a pivotal mediator through which T2D differentially modulates scar formation, reflecting fundamental disparities in immune microenvironment composition and cellular dynamics between the two scar types. The opposing expression trajectories of CD4^+^ NT in HS and keloids imply that T2D may influence divergent pathological outcomes by regulating their differentiation and activation trajectories.

As pivotal mediators of adaptive immunity, CD4^+^ NT regulate local inflammation, promote tissue regeneration, and contribute to fibrotic remodeling by differentiating into distinct effector lineages such as Th1, Th2, Th17, and Treg subsets. Fluctuations in their activation states and abundance can directly impact the cytokine milieu, fibroblast activity, and collagen deposition processes. Previous studies have shown that CD4^+^ NT can differentiate into Th17 cells to promote IL-17 production, a pro-inflammatory and pro-fibrotic cytokine that exacerbates hepatic fibrosis[38]. Li *et al*[39] through single-cell analyses of normal and keloid skin, demonstrated distinct immune cell cluster distributions via deep-learning-based dimensionality reduction (tSNE), with CD4^+^ NT significantly reduced in keloid tissues – consistent with our findings. Another study using the ssGSEA algorithm reported an increased infiltration of effector memory CD4^+^ T cells in keloids compared to normal skin[40].

Further cell–cell communication analysis revealed extensive ligand–receptor interactions between CD4^+^ NT and multiple scar-associated structural cells, including endothelial cells, fibroblasts, and dendritic cells. Among these, the INS–INSR emerged as the most active axis in both HS and keloid tissues, suggesting it may represent a key molecular link between glucose metabolism dysregulation and immune microenvironment remodeling. Within the scar microenvironment, insulin activates the INSR–PI3K–Akt–mTOR signaling cascade in CD4^+^ NT, enhancing glucose uptake and glycolytic flux while lowering the metabolic threshold for differentiation from naive to effector states and augmenting functional output. Loss of endogenous INSR expression in T cells impairs both glycolytic and mitochondrial metabolism as well as effector functionality^[41,42]^. This axis establishes a self-reinforcing “receptor–pathway–metabolism–function” loop through upregulation of GLUT1, which is indispensable for the metabolic reprogramming, proliferation, and survival of activated CD4^+^ T cells[43]. mTORC1/HIF-1α further drives glycolytic gene expression and effector programs, coupling them with migration-related processes. In tissue-like three-dimensional collagen matrices, T-cell motility relies more heavily on mitochondrial oxidation of glucose and glutamine, defining a context-dependent boundary for metabolic control of migration^[44,45]^. In pathological scars, predominant Th2 cytokine signaling (IL-4/IL-13) directly promotes fibroblast-mediated collagen synthesis, while insulin synergizes with CTGF to enhance collagen production, thereby coupling immune–metabolic amplification with matrix-remodeling pathways that drive fibrotic reconstruction^[46–48]^. In endothelial cells, insulin activates the INSR–PI3K–Akt pathway and phosphorylates eNOS, leading to increased nitric oxide (NO) production, vascular relaxation, and enhanced endothelial survival and repair – mechanisms validated in both human and experimental endothelial models^[49,50]^. Concurrently, insulin promotes endothelial migration, proliferation, and in vitro tube formation, exhibiting pro-angiogenic activity[51]. Angiogenic sprouting is profoundly dependent on metabolic regulation: PFKFB3 upregulation drives glycolysis in tip cells, supporting lamellipodia formation and branching; pharmacologic or genetic inhibition of PFKFB3 (e.g., with 3PO) suppresses aberrant sprouting and tip-cell competitiveness^[52,53]^. Additionally, endothelial fatty acid oxidation via CPT1A supplies carbon for DNA synthesis to sustain proliferation, and CPT1A loss results in defective sprouting rather than energy depletion[54]. Collectively, these metabolic programs, together with VEGF–Dll4/Notch-mediated tip–stalk cell interactions, may contribute to vascular patterning and help establish both signaling and metabolic foundations for “vascular–matrix” remodeling during the scar maturation phase^[55,56]^. This study, for the first time, provides single-cell level evidence demonstrating that metabolic aberrations modulate CD4^+^ NT states and their intercellular communication with scar-resident cells, collectively driving the formation and heterogeneity of pathological scars. These findings underscore the critical role of metabolic–immune crosstalk in the pathogenesis of cutaneous fibrotic disorders.

Through comprehensive integration of multi-omics data, this study identified three pivotal DEGs (GPR35, TMEM91, and ZBTB32) closely associated with CD4^+^ NT, exhibiting diametrically opposed expression patterns between HS and keloids. These findings suggest that these genes may serve as central regulatory mediators through which T2D differentially influences the pathogenesis of distinct scar subtypes, exerting bidirectional effects on local immune modulation and fibrotic remodeling. GPR35, an immune-related GPCR predominantly coupled to Gi, has been implicated in the regulation of inflammation and in neutrophil/leukocyte migration and extravasation, suggesting that a GPR35-mediated cAMP–motility signaling axis can tune immune-cell activation thresholds and engage metabolic programs encompassing glycolysis, lipid metabolism, and redox homeostasis^[57–60]^. In parallel, GPR35 functions as a metabolically responsive GPCR activated by endogenous metabolites such as kynurenic acid (KYNA), coupling to Gi to modulate inflammatory phenotypes and metabolic wiring; under metabolic dysregulation (e.g., alterations in the tryptophan–kynurenine pathway in T2D), the KYNA–GPR35 axis may reshape immune-cell trafficking/tissue residency and activation thresholds, secondarily influencing T-cell polarization and the inflammatory-to-repair tempo in the wound bed, thereby affecting ECM deposition and fibrotic progression. Notably, GPCR signaling within fibroblasts is recognized as a critical regulatory layer with anti- and pro-fibrotic consequences^[61,62]^. GPR35, a G protein–coupled receptor, has recently been implicated in inflammatory regulation and tissue repair. Kim *et al*[63] reported that lodoxamide, a potent GPR35 agonist, ameliorated liver fibrosis in murine models, whereas administration of a selective GPR35 antagonist abrogated its antifibrotic effect, indicating a potential role of GPR35 in scar tissue fibrosis. ZBTB32, a POZ/BTB zinc-finger transcription factor, cooperates with Blimp-1 during T-cell activation to restrain excessive effector responses and shape memory formation, thereby occupying an upstream position in the transcriptional network that programs cellular metabolism^[64,65]^. As a BTB-ZF factor, ZBTB32 is transiently induced during antiviral responses to limit T-cell effector function and memory development, implying that by modulating activation and memory programs it can influence metabolic expenditure and resource allocation. Broader evidence across the ZBTB family indicates tight coupling between transcriptional control, mitochondrial activity, and glucose metabolism, supporting a testable “ZBTB32–metabolism–function” axis; by altering the immunokine repertoire, this axis may secondarily regulate fibroblast activity and collagen deposition^[64,66]^. TMEM91 is largely assigned to membrane trafficking/endocytosis and autophagy networks; the autophagy–vesicular transport axis is closely linked to metabolic switching in immune cells, non-canonical secretion, and extracellular matrix regulation[67]. We therefore cautiously position TMEM91 as a candidate node at the immune–metabolic interface, hypothesizing that it may modulate fibrotic phenotypes by altering vesicle flux and the secretome; however, causal evidence in primary T cells and fibrosis remains to be established through functional studies. Moreover, pseudotime trajectory analysis revealed divergent temporal expression dynamics of these key genes within CD4^+^ NT between HS and keloids, underscoring distinct activation and silencing kinetics throughout pathological progression. This highlights significant heterogeneity in immune regulatory networks underlying scar formation and offers potential temporal and molecular targets for precise therapeutic intervention, thereby providing novel theoretical foundations for mechanistic exploration and translational applications.

The intersecting-gene analyses collectively suggest that T2D may exert divergent effects on HS and keloids through an “immunometabolic” reprogramming axis. On one hand, the “T2D–HS” intersecting gene set is predominantly enriched in focal adhesion, ECM–receptor interaction, efferocytosis, and leukocyte migration pathways, delineating a signature characterized by inflammatory cell trafficking and localized tissue repair. This pattern implies that, in the context of T2D, HS may resemble a focal hyperplastic response driven by a relatively mild shift in the inflammation–repair equilibrium. On the other hand, the “T2D–keloid” intersecting gene set shows marked enrichment in PI3K–Akt signaling, insulin resistance, AGE–RAGE signaling, HIF-1 signaling, and extracellular matrix organization, together with pronounced enrichment of fatty acid metabolism and oxidative phosphorylation, indicative of a “high-fibrotic + metabolically active state” phenotype orchestrated by metabolic imbalance, hypoxic responses, and sustained ECM remodeling. Concomitant enrichment of PI3K–Akt, VEGF, and multiple cytokine receptor–related pathways alongside glycolysis, fatty acid metabolism, and oxidative phosphorylation further supports the concept that immune signaling and metabolic circuitry are integrated into a unified “immunometabolic axis.” In parallel, the opposing expression patterns of CD4^+^ Naive T cell–associated genes such as GPR35, TMEM91, and ZBTB32 between HS and keloids suggest that T2D may differentially imprint systemic metabolic dysregulation onto the local microenvironments of distinct scar subtypes by reshaping immunometabolic states. Nonetheless, this hypothesis requires validation through dedicated mechanistic and functional experiments.

Moreover, this study uncovers the potential roles of metabolic regulatory genes (GPR35, TMEM91, and ZBTB32) in orchestrating metabolic reprogramming of CD4^+^ NT within HS and keloids. We observed that, in keloid tissue, CD4^+^ NT exhibiting high expression of these genes were significantly enriched in multiple pathways associated with elevated metabolic activity, suggesting a metabolically activated and functionally primed state. Previous studies have established that metabolic reprogramming is a pivotal determinant of T cell activation, differentiation, and tissue adaptation, particularly under chronic inflammatory and tissue remodeling conditions[68]. Hass *et al*[69] revealed that CD4^+^ T cells detect extracellular lactate through the SLC5A12 transporter, which compromises their motility and promotes their accumulation at inflamed tissues, thus sustaining chronic inflammation. Within the inflammatory microenvironment, T cells are exposed to a spectrum of metabolic stressors, including hypoxia[70], depletion of glucose and glutamine[71], lactate accumulation[72], and elevated ROS levels^[73,74]^. Under such conditions, metabolic flexibility becomes a critical determinant of T cell fate, potentially initiating the transition from a quiescent to an effector phenotype and fueling persistent inflammation and fibrosis. In contrast, CD4^+^ NT derived from HS tissues, despite their increased abundance, exhibited reduced enrichment and expression across metabolic pathways, indicative of a relatively quiescent metabolic state. This divergent metabolic profile likely reflects fundamental differences in the inflammatory burden, tissue repair kinetics, and immune microenvironmental homeostasis between the two scar phenotypes. Notably, the metabolic features described above align with transcriptomic findings of scar-type-specific pathway enrichment, reinforcing the pivotal role of metabolic reprogramming in pathological scar development. As potential metabolic regulatory hubs, GPR35, TMEM91, and ZBTB32 may function as key modulators driving the functional state transitions of CD4^+^ NT.

These findings offer mechanistic insights that may inform future clinical decision-making and the development of targeted therapeutic interventions. In patients with T2D, the differential impact on distinct scar subtypes should be considered in risk assessment, particularly in the prevention and treatment of HS, where both metabolic regulation and immune modulation warrant integrated therapeutic consideration. CD4^+^ NT and the identified key regulatory genes represent promising candidates for targeted intervention, while intercellular communication pathways, such as the INS–INSR axis, may inform future pharmacological development. The interdisciplinary approach combining single-cell omics with MR exemplifies a paradigm for deciphering the mechanistic underpinnings of complex diseases within the framework of precision medicine.

This study not only delineates the bidirectional mechanisms by which T2D influences HS and keloids but also outlines potential pathways for clinical translation. From a therapeutic perspective, drug repurposing and target druggability hold significant promise: existing metabolic–immune modulators, such as GLP-1 receptor agonists, may be repurposed to prevent hypertrophic scarring in diabetic patients, whereas GPR35 regulators represent potential antifibrotic strategies, supported by evidence that GPR35 agonists can attenuate hepatic fibrosis[63]. Equally critical is the development of biomarkers: the frequency and metabolic state of CD4^+^ NT emerge as candidate prognostic indicators of scar heterogeneity, facilitating patient stratification and treatment monitoring. Looking ahead, we envision a stepwise translational roadmap: (1) external validation of genetic and single-cell findings across multi-ethnic cohorts; (2) mechanistic studies in diabetic scar models and T cell–endothelial cell co-culture systems; and (3) integration of candidate biomarkers with targeted interventions in early-phase clinical trials. Collectively, these efforts may advance the translation of genetic and multi-omics evidence into precision medicine strategies for pathological scarring.

Despite integrating genetic causal inference with multi-omics analyses to enhance depth and robustness, this study has several limitations. First, although MR can mitigate confounding and strengthen causal inference, it is constrained by the availability and validity of IVs; relevant variants for pathological scarring may be missed, and horizontal pleiotropy or measurement error cannot be fully ruled out. Second, the MR framework did not explicitly incorporate epigenetic regulation (e.g., DNA methylation, histone modifications, non-coding RNAs), which profoundly shapes gene expression and immune–metabolic reprogramming but is not fully captured by germline variation. Third, the genetic datasets were predominantly derived from European-ancestry populations, limiting generalizability to other ancestries. Fourth, most single-cell datasets were obtained from public repositories with modest sample sizes and are inevitably affected by batch effects and inter-individual heterogeneity, warranting cautious interpretation; validation in large, multi-center clinical cohorts is needed to improve robustness and representativeness. Finally, this study aimed to delineate putative pathways by which T2D influences scar phenotypes along the immune–metabolic axis. Islet scRNA-seq data from individuals with T2D were used not to claim inter-tissue equivalence, but to anchor disease-related immune–metabolic programs and to assess whether perturbations in INS–INSR signaling and T-cell metabolic configuration mirror those observed in cutaneous scars. Cross-tissue inference is inherently constrained by microenvironmental and lineage-specific differences – the pancreatic islet and the wound bed differ markedly in stromal, vascular, and cytokine contexts – so any concordance should be considered hypothesis-generating rather than evidence of organ-wide generalizability. Moreover, the absence of paired pancreatic and skin samples from the same individuals precludes subject-level validation. At the functional level, evidence supporting the roles of key genes (*GPR35, ZBTB32*, and *TMEM91*) and implicated signaling pathways remains limited. Future work should integrate targeted *in vitro* and *in vivo* perturbation to delineate mechanism and evaluate translational potential, thereby bridging fundamental discovery with clinical application.

## Conclusion

Drawing on convergent multi-omics evidence, we advance and substantiate the working hypothesis that T2D exerts opposing causal effects on HS versus keloids, indicating distinct mechanistic heterogeneity between these pathological scars. By integrating single-cell and bulk transcriptomes, we show that T2D modulates the abundance and metabolic programs of CD4^+^ NT, thereby reshaping the immune microenvironment and altering intercellular communication exemplified by the INS–INSR axis. In parallel, we identify *GPR35, TMEM91*, and *ZBTB32* as salient genes, implicating CD4^+^ NT metabolic reprogramming as a central conduit linking systemic metabolic dysregulation to pathological scarring and nominating putative targets for precision intervention.

## Data Availability

All data used in this study are available upon reasonable request. The analysis code has been deposited in an anonymized GitHub repository (link to be provided after acceptance).
